# Resuscitative Endovascular Balloon Occlusion of the Aorta for Blood Control in Lumbar Spine Tumor Resection Surgery: A Technical Note

**DOI:** 10.1111/os.13048

**Published:** 2021-06-04

**Authors:** Yong‐jie Zhao, Xin‐chong Du, Xiao‐qiang Deng, Hao Zhang, Hao‐ran Zhang, Rui‐qi Qiao, Jing‐yu Zhang, Yong‐cheng Hu

**Affiliations:** ^1^ Tianjin Medical University Tianjin Medical University Tianjin China; ^2^ Binzhou Medical University Hospital Binzhou Medical University Hospital Binzhou China; ^3^ Tianjin Hospital, Department of Bone Tumor and Soft Tissue Oncology Tianjin Hospital Tianjin China

**Keywords:** Aortic occlusion, Balloon, Blood control, Hemorrhage, Lumbar spine

## Abstract

**Objectives:**

To describe the technique of the aorta balloon occlusion, and evaluate the blood loss in lumbar spine tumor surgery assisted by aortic balloon occlusion, and to observe the balloon‐related complications.

**Methods:**

Six patients with lumbar spine tumor underwent resuscitative endovascular balloon occlusion of the aorta prior to tumor resections in our institution between May 2018 to January 2021. Medical records including demographic, diagnosis, tumor location, surgical approach, intraoperative blood loss, surgical duration, and perioperative balloon‐related complication were evaluated retrospectively.

**Results:**

This series included four males and two females, with a median age of 50 years (range 22 to 69). Of these, three primary tumors were plasmacytoma, giant cell tumor of bone, and osteosarcoma, while recurrence of undifferentiated pleomorphic sarcoma (UPS), recurrence of giant cell tumor of bone (GCT), and metastatic thyroid cancer were diagnosed in cases 1, 6, and 2, respectively. L_2_ was involved in cases 1 and 5. L_3_ was involved in case 6. L_4_ was involved in case 2, 3, and 6. L_5_ was involved in case 4. One‐stage total en bloc resection surgery (TES) was accomplished in all patients; of this series, signal anterior approach was conducted in case 1, signal posterior approach was utilized in cases 2, 3, and 6, while combined anterior and posterior approach was performed in cases 4 and 5. The median intraoperative blood loss was 1683 mL and ranged from 400 to 3200 mL with a median surgical duration of 442 min and a range from 210 to 810 min. During the perioperative period, no serious balloon‐related complications occurred.

**Conclusions:**

Endovascular balloon occlusion of the aorta successfully controls intraoperative exsanguination, contributing to a more radical tumor resection and a low rate of tumor cell contamination in lumbar tumor surgery.

## Graphical abstract

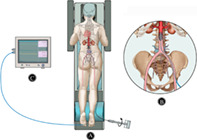

Schematic diagram of the patient with a tumor involved to level L_4_ had resuscitative endovascular balloon occlusion of the aorta intraoperatively. (i) The patient was transferred to the interventional radiology unit and placed in supine position. Abiding by the Seldinger technique, a balloon dilation catheter (BDC) was inserted to the designated location and then shifted to prone position. (ii) The expected position of the balloon was cephalad to abdominal aortic bifurcation but caudal to the renal arteries (zone III). After the balloon inflated, blood flow to the distal aorta was stopped during the subsequent tumor resection. (iii). During orthopaedic oncology procedure, occlusion of abdominal aorta was identified by the disappearance of dorsal pedis arteries and pulse oximeter oxygen saturation (SpO_2_) signals from toe.

## Introduction

The spine is the most common region of osseous metastasis, even though the primary tumors occupy less than 5% of bone neoplasms^1^. The lumbar spine is involved in 20% of spinal metastases^2^ and being accompanied by maximum exsanguination in site of spinal operation^3^. Chen *et al*.^4^ performed a meta‐analysis of 18 studies related to spinal tumor surgery and reported that up to 12% of catastrophic blood loss (>5000 mL) occurred in spinal metastasis surgery, which was associated with increased mortality. In addition, extensive bleeding can produce intraoperative tumor cell contamination resulting in local recurrence^5^. Therefore, controlling hemorrhage is critical for surgical excision of lumbar spine tumors.

Several interventions including deliberate hypotension, the use of antifibrinolytic and preoperative embolization, has been studied in an attempt to minimize the intraoperative bleeding^4^, ^6^, ^7^. Of these methods, super‐selective embolization was considered an optimal technique for blood control in spinal tumor surgery. Tan *et al*.^8^ conducted a retrospective review of 221 patients who underwent the spinal oncologic surgery. Median blood loss was lower in 48 patients who received preoperative embolization compared to 173 patients who did not. They concluded that preoperative spinal tumor embolization was effective in reducing hemorrhage. Hong *et al*.^9^ performed 18 preoperative embolization in metastatic spinal cord compression (MSCC) patients and concluded that preoperative embolization was relatively safe and effective for controlling intraoperative blood loss.

Though the use of tranexamic acid could significantly decrease transfusions during the surgery for spinal tumors, it was not of benefit for less intraoperative blood loss^10^. A meta‐analysis of 30 randomized controlled trials reported that the safety of deliberate hypotension for orthopaedic surgery was still unclear^6^. In addition, two recent studies concerning embolization in surgical treatment of spinal metastases validated that preoperative embolization could not reduce intraoperative exsanguination, though it did reduce the surgical duration^11^, ^12^. Therefore, the effect of these techniques in blood loss management remains controversial.

Resuscitative endovascular balloon occlusion of the aorta (REBOA) is a technique to directly obstruct blood flow into distal aorta assisted by inflated balloon. Previous studies have shown that the use of the aorta balloon successfully reduced intraoperative hemorrhage in sacropelvic tumor surgeries^5^, ^13^, ^14^, ^15^, ^16^, ^17^, ^18^, ^19^. To our knowledge, no reports have investigated the use of aorta balloon catheter in lumbar spine tumor surgery. Therefore, we attempted to perform resuscitative endovascular balloon occlusion of the aorta prior to surgery in patients with lumbar spine tumors, aiming to: (i) describe the technique of the aorta balloon occlusion; (ii) evaluate the blood loss in lumbar spine tumor surgery assisted by aortic balloon occlusion; and (iii) discuss the balloon‐related complications.

## Methods

Between May 2018 to January 2021, REBOA was performed on six patients with lumbar spine tumors before the operation. This series included four males and two females, with a median age of 50 years (range 22 to 69). Three primary tumors were plasmacytoma, giant cell tumor of bone, and osteosarcoma, while recurrence of undifferentiated pleomorphic sarcoma (UPS), recurrence of giant cell tumor of bone (GCT), and metastatic thyroid cancer were diagnosed in cases 1, 6, and 2, respectively. L_2_ was involved in cases 1 and 5. L_3_ was involved in case 6. L_4_ was involved in cases 2, 3, and 6. L_5_ was involved in case 4. One‐stage total en bloc resection surgery (TES) was accomplished in all patients. Of this series, signal anterior approach was conducted in case 1, signal posterior approach was utilized in cases 2, 3, and 6, while combined anterior and posterior approach was performed in cases 4 and 5. The details were summarized in Table 1. The surgical indications included neurologic deficits, mechanical instability, intractable pain. Case 3 underwent dynamic contrast‐enhanced magnetic resonance imaging perfusion (DCE‐MRI), and the other five cases obtained computed tomographic angiography (CTA). All patients were validated as having no vascular malformation, aneurysm, or unstable plaque.

**TABLE 1:** Patient characteristics

Case number	Gender	Age (years)	Diagnosis	Tumor location	Approach
1	Male	22	Recurrence of UPS^*^	L_2_	Anterior
2	Female	55	Metastasis of thyroid cancer	L_4_	Posterior
3	Male	69	Plasmacytoma	L_4_	Posterior
4	Female	43	Giant cell tumor of bone	L_5_	Combination
5	Male	56	Osteosarcoma	L_2_ and Pelvic	Combination
6	Male	44	Recurrence of GCT^†^	L_3_ and L_4_	Posterior

^*^ UPS: undifferentiated pleomorphic sarcoma.

^†^ GCT: giant cell tumor of bone.

## Imaging Study

The levels of lumbar vertebra involved by tumor on preoperative plain radiography (PR) were from L_2_ to L_5_. Of these, case 5 involved both L_2_ and pelvis, and case 6 had a recurrence of GCT in the level of L_3_ and L_4_. Computerized tomography (CT) demonstrated vertebral osteolytic destruction in five patients and local kyphotic deformity due to pathological collapse of L_4_ in case 6. Preoperative magnetic resonance imaging (MRI) showed vertebral lesions with a low signal on T1‐weighted image and a high signal on T2‐weighted image. Of six patients, axial and sagittal T1‐weighted MR images depicted cord compression due to osteolytic destruction in four patients, and Gd‐enhanced T1‐weighted MR images revealed a contrast‐enhancing tumor involving the L4 with extension into the spinal canal, compressing the right L_4_ nerve roots in case 2. In patients with primary tumors, emission computed tomography (ECT) depicted the concentration of radioactivity was limited in the vertebral body without distal metastasis.

## REBOA Technique

**Fig. 1:**
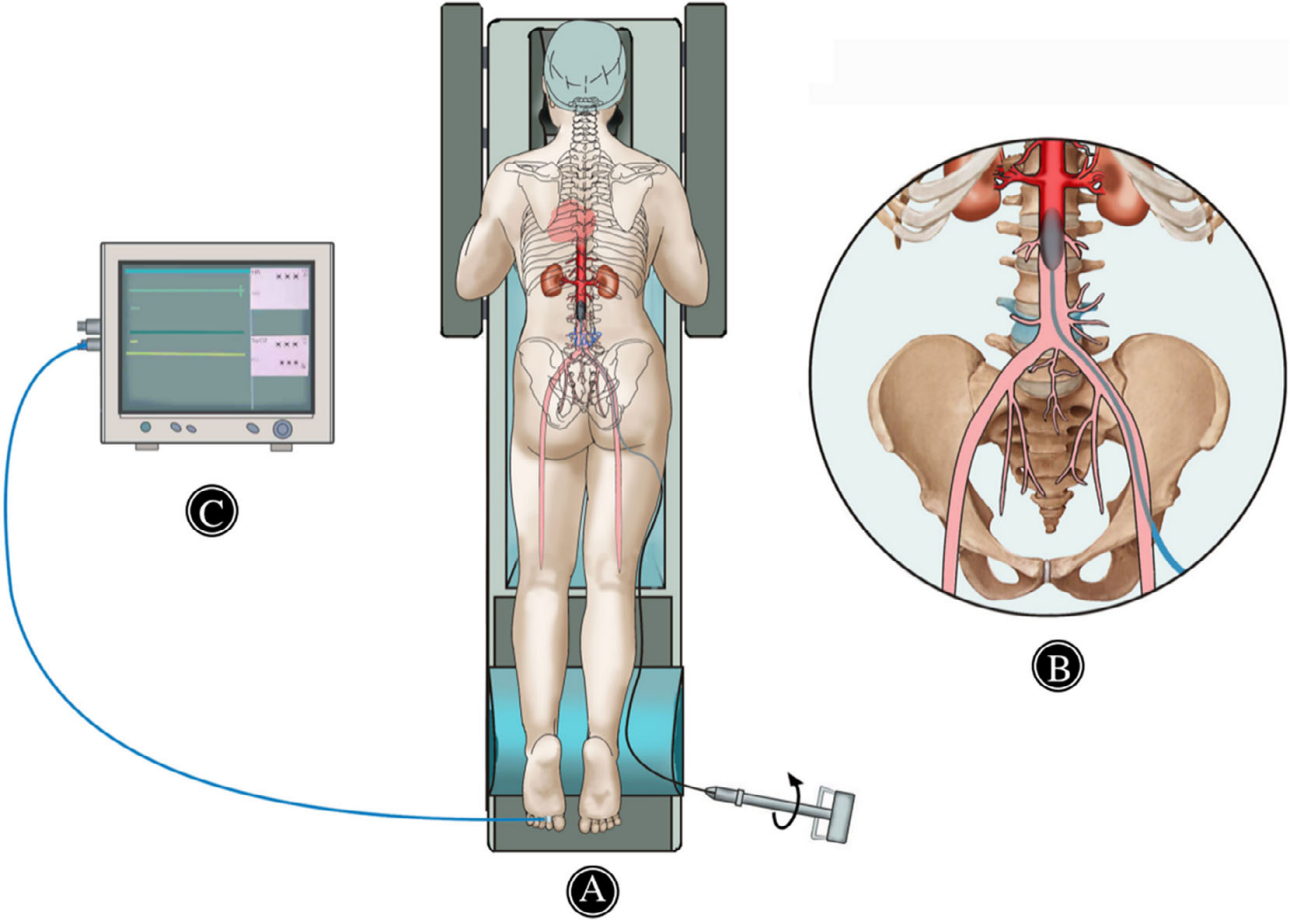
Schematic diagram of a patient with a tumor involved to level L_4_ obtained resuscitative endovascular balloon occlusion of the aorta intraoperatively. (A) The patient was transferred to the interventional radiology unit and placed in supine position. Abiding by the Seldinger technique, a balloon dilation catheter (BDC) was inserted into the designated location and then shifted to prone position. (B) The expected position of the balloon was cephalad to abdominal aortic bifurcation but caudal to the renal arteries (zone III). After the balloon inflated, blood flow to the distal aorta was stopped during the subsequent tumor resection. (C) During orthopaedic oncology procedure, occlusion of abdominal aorta was identified by the disappearance of dorsal pedis arteries and pulse oximeter oxygen saturation (SpO_2_) signals from toe.

On the day of surgery, the patient was first transferred to the interventional radiology unit and placed in supine position. Both groin regions were sterilized with iodine solution. After adequate local anesthesia, abiding by the Seldinger technique^20^, the target femoral artery was punctured with an arterial catheter needle and a guidewire was introduced, then an 8F percutaneous introducer sheath with dilator (Merit Medical) was inserted (Fig. 1A). The diameter of abdominal aorta was measured, and the location of renal arteries was determined under the abdominal aortography.

A balloon dilation catheter (BDC) with diameter 1–2 cm larger than the abdominal aorta dimension was selected. Before being inserted, the BDC was conventionally checked with contrast medium to ensure its integrity and patency and was advanced over the wire to its designated location. The expected location of the balloon was maximumly cephalad to abdominal aortic bifurcation but caudal to the renal arteries (Fig. 1B). Then the balloon was filled with contrast medium to pre‐occlude the abdominal aorta. Complete occlusion was identified when blood flow into aorta caudal to the balloon disappeared and the amount of contrast medium was recorded precisely. After the balloon was deflated, the end of the catheter was fixed firmly with a skin suture to prevent any displacement.

Prior to orthopaedic operation, the balloon was inflated again with the same amount contrast medium. Intraoperative abdominal aorta occlusion was identified by the disappearance of dorsal pedis arteries and pulse oximeter oxygen saturation (SpO_2_) signals from toe (Fig. 1C). In these series, we usually deflated the balloon completely for 20 min after each 90 min occlusion and stopped operating intermittently. Meanwhile, the urine volume and blood pressure were obliged to be monitored in operation.

## Orthopaedic Oncology Surgery Technique

**Fig. 2:**
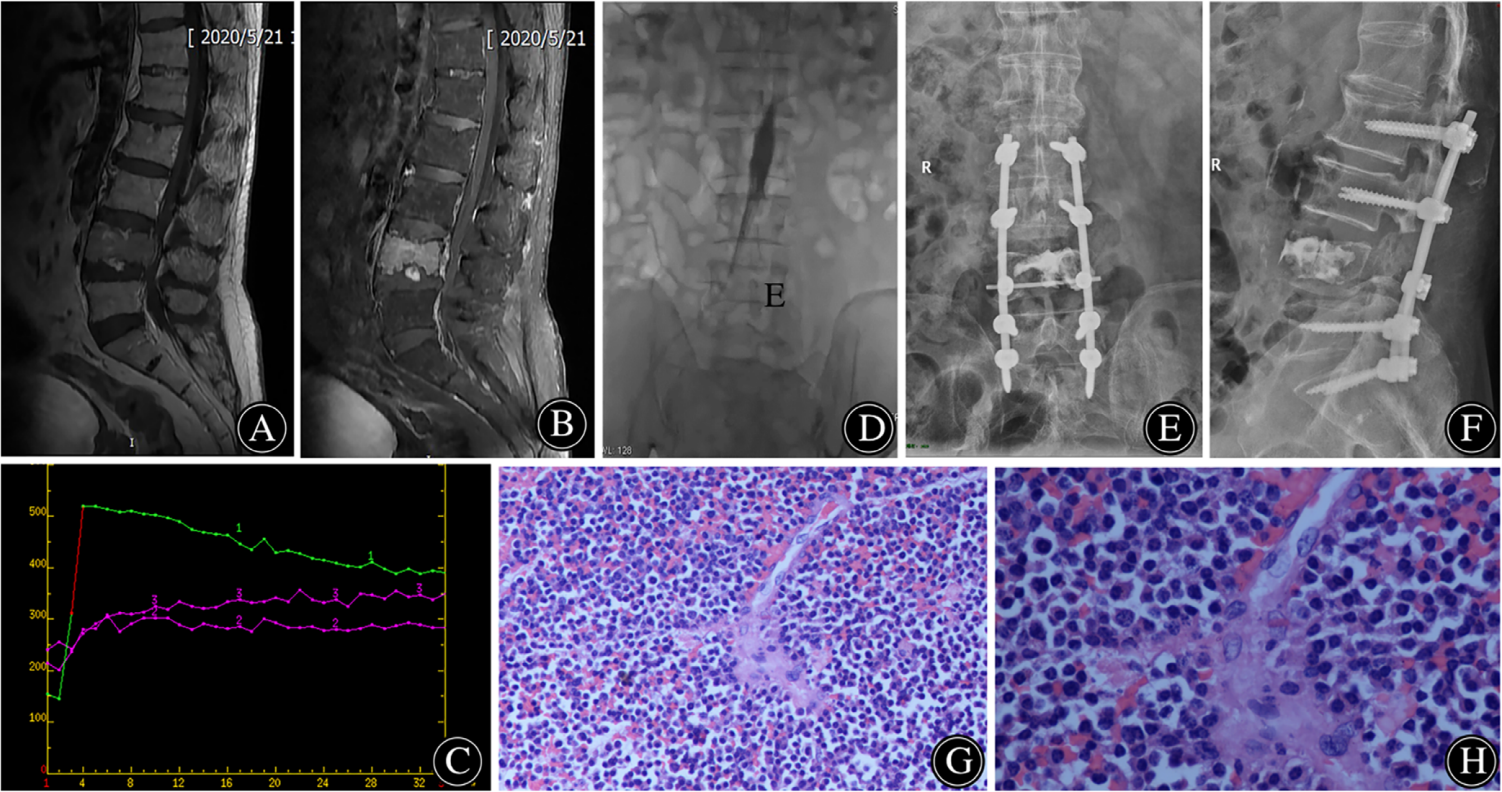
A 69‐year‐old male with lumbar spine tumor. (A, B) Preoperative magnetic resonance (MR) showed a low signal on T1‐weighted image and a high signal on T2‐weighted image in level L_4_. (C) Preoperative DCE‐MRI was performed and the average time‐signal intensity curve depicted increased blood flow to the lesions. (D) Intraoperative fluoroscopy revealed the position of balloon catheter cephalad to L_4_ vertebra and caudal to the renal arteries. (E, F) After tumor resection, spinal stability was reconstructed with pedicle screw‐rod system. (G, H) A plasmacytoma was diagnosed histologically by paraffin sections postoperatively.

The patient was transferred to the radiolucent operating table and shifted to prone position. Once placed in the correct aortic zone, the balloon minimized blood flow to the lumbar spine tumors. As a result of a bloodless surgical field, the distinct edge of the tumor and lumbar nerve roots were easily exposed and en bloc resection was performed in six patients. Then, decompression of the spinal cord, reconstruction and stabilization of the lumbar spine were conducted individually. After surgery, the balloon catheter was subsequently removed and the femoral arterial puncture site was closed with perclose proglide (Abbott Vascular) (Fig. 2).

## Observational Indicators

### Intraoperative Blood Loss

The intraoperative blood loss was an indicator to evaluate the hemorrhage during the operation, which was calculated by measuring the volume of mechanical suction and absorption of gauzes (1 g = 1 mL). Less intraoperative blood loss predicted lower rate of tumor cell contamination and more hemodynamic stability.

### Operative Duration

The operative duration was an indicator to evaluate the time of whole procedure. Due to the fact that balloon catheter was performed under local anesthesia in this series and no blood loss occurred during the procedure, the reasonable operative duration was estimated from incision to wound closure. Short operative duration was associated with low rate of wound infection after operation.

### Balloon‐related Complication

As an index reflecting safety of aortic balloon occlusion, the balloon‐related complication was recorded from the moment of balloon insertion, mainly including intraoperative hypertension, cardiac events, perioperative acute kidney injury, and vascular events.

## Results

### Intraoperative Blood Loss

The median intraoperative blood loss was 1683 mL and ranged from 400 to 3200 mL. The patient with recurrence of UPS (case 1) underwent single anterior approach operation with bleeding of 400 mL. While the patient with giant cell tumor of bone (case 4) underwent tumor resection and spinal stability reconstruction through one‐staged combined posterior and anterior approach, 3200 mL blood was lost during the whole procedure.

### Operative Duration

The median operative duration was 442 min and ranged from 210 min to 810 min. The female with giant cell tumor of bone (case 4) underwent tumor resection and spinal stability reconstruction through combined posterior and anterior approach at one stage. Due to obese abdomen, transperitoneal resection was required for this patient, and the procedure consumed nearly 810 min.

### Balloon‐related Complications

In six patients, no shock and balloon rupture occurred when the balloon was inflated and deflated during the surgery. There was no hematoma formation, pseudoaneurysm, thrombosis, or kidney injury perioperatively.

All patients were ambulatory on discharge. Except loss of follow‐up in case 1 after being discharged from hospital, the remaining five patients were alive with follow‐up for a mean of 24.9 months (range, 2–34 months). There were no cases of screw or instrumentation failure on postoperative radiograph. Case 4 had a local recurrence in L5 at 1 year postoperatively and was treated with Dinosemer.

## Discussion

### Anatomical Basis for REBOA Feasible in Lumbar Spine Tumor Surgery

In normal adults, abdominal aorta bifurcates into the right and left renal arteries and common iliac arteries at the approximate level of the second and fourth lumbar vertebra, respectively. That means the more than 6 cm distance between them permits a sufficient space to place an inflated balloon. In addition, due to anastomotic branch between inferior mesenteric artery and superior mesenteric artery, the intestinal blood supply was not occluded even the balloon position is proximal to inferior mesenteric artery. The balloon occlusion technique is theoretically applicable in the lumbar spine oncology surgery when the tumor is located caudal to the renal arteries.

### Effects of REBOA in Lumbar Spine Tumor Surgery

Lumbar spine tumor resection is associated with large intraoperative blood loss. Gao *et al*.^21^. reported that the bleeding originated from the dilated epidural venous plexus, paraspinal blood vessels, uninvolved bone, and soft tissues aside from the hypervascular tumor itself. Unclear surgical field due to hemorrhage increases the rate of nerve and adjacent blood vessel damage. Moreover, indistinct tumor boundary and tumor cell contamination caused by massive bleeding easily leads to tumor relapse. Just like a tourniquet used on the extremities, the aortic balloon can directly decrease blood flow to the distal aorta during the tumor resection. As a result, the surgical margin of the tumors and lumbar nerve roots can be identified clearly, contributing to a more radical tumor resection without contamination in the operating site and iatrogenic neural injury. Decreasing operative duration is the secondary benefits. Luo *et al*.^5^ described that control of bleeding rather than removal of the tumor consumes a considerable part of the operative duration. In this series, the operative duration was reduced owing to clear visualization of the surgical field and accessibility of lesions under the aorta balloon occlusion. In particular, the minor impact on arterial blood supply to the adjacent normal structures after balloon deflation is another advantage of REBOA technique.

### Technotes of REBOA

Prior to operation, cardiac and systemic coagulation function of patients should be evaluated, due to the increase in afterload and change in coagulable condition after the balloon placement. Be noted that digital subtraction angiography (DSA) or CTA is conventionally used to exclude the vascular malformation, aneurysm, and unstable plaque. As a noninvasive measurement for hemodynamic characteristics, DCE‐MRI is helpful to allow surgeons to determine if preoperative REBOA is required to decrease intraoperative hemorrhage^22^.

Though REBOA is a potential alternative for extensive blood loss control, prolonged occlusion of the aorta may produce ischemia–reperfusion injury. Until now, a maximal safety occlusion time has not yet reached a consensus. A median occlusion time of 55.2 min was reported in 19 studies included 223 patients undergoing zone III occlusion^23^. Another study indicated that patients with sacral tumors received one single continuous occlusion duration of less than 90 min and had minor damage and recovered to normal levels after 4 h^15^. However, the replantation of severed limb disclosed that the limb can endure more than 6 h ischemic time^24^. In this study, the longest single persistent balloon occlusion duration was up to 90 min, and no ischemic necrosis of lower extremity occurred. To reduce occlusion time, balloon inflated after exposure of the tumor was suggested. Intermittent occlusion was another recommended solution to decrease the ischemic time of the limb. Unfortunately, it usually causes hemodynamic instability even circulatory collapse in old patients; as a result, deflation of the balloon should be executed very slowly. In addition, the intermittent duration and safety times for occlusion remain controversial.

The balloon‐related complications mainly include hematoma formation, pseudoaneurysm, thrombosis, and a 6% rate of vascular events occurred in a large series of 911 patients undergoing coronary revascularization^25^. Another meta‐analysis of 89 studies described a less than 5% rate of iatrogenic injury related to REBOA^23^. Interestingly, injury to the vascular wall of the artery by the inflated balloon has not been found. In our six cases, no serious vascular complications occurred. Although renal injury was a potential severe complication caused by aortic balloon occlusion, according to our experience, there is no need to be excessively worried because the balloon will not transfer to the position cephalad to the renal arteries under the hyperbaric blood flowing once the balloon catheter is in the correct location. Only one research reported two acute kidney injuries that occurred in 56 patients with sacral tumors involving the lower lumbar spine by aortic balloon occlusion^18^. The most likely reason was that the balloon catheter was placed cephalad to the renal arteries before the orthopaedic oncology surgery.

To date, our study was the first attempt to use REBOA technique in management of blood loss during lumbar spine tumor surgery and ongoing research will provide higher quality data in the coming years.

## Conclusion

Endovascular balloon occlusion of the aorta successfully controls intraoperative exsanguination, contributing to a more radical tumor resection and a low rate of tumor cell contamination in lumbar tumor surgery.

### An Authorship Declaration

(i) All authors meet the authorship criteria according to the latest guidelines of the International Committee of Medical Journal Editors, and (ii) all authors are in agreement with the manuscript.
